# Modified Kibria-Lukman (MKL) estimator for the Poisson Regression Model: application and simulation

**DOI:** 10.12688/f1000research.53987.2

**Published:** 2021-12-14

**Authors:** Benedicta B. Aladeitan, Olukayode Adebimpe, Adewale F. Lukman, Olajumoke Oludoun, Oluwakemi E. Abiodun

**Affiliations:** 1 Department of Physical Sciences, (Mathematics) Landmark University, Omu-Aran, Kwara State, +234, Nigeria; 2 SDG 13 (Climate Action), Landmark University, Omu-Aran, Kwara State, Nigeria

**Keywords:** Linear regression model, generalized regression model, Ridge estimator, Liu estimator, KL estimator.

## Abstract

**Background:** Multicollinearity greatly affects the Maximum Likelihood Estimator (MLE) efficiency in both the linear regression model and the generalized linear model. Alternative estimators to the MLE include the ridge estimator, the Liu estimator and the Kibria-Lukman (KL) estimator, though literature shows that the KL estimator is preferred. Therefore, this study sought to modify the KL estimator to mitigate the Poisson Regression Model with multicollinearity.

**Methods:** A simulation study and a real-life study was carried out and the performance of the new estimator was compared with some of the existing estimators.

**Results: **The simulation result showed the new estimator performed more efficiently than the MLE, Poisson Ridge Regression Estimator (PRE), Poisson Liu Estimator (PLE) and the Poisson KL (PKL) estimators. The real-life application also agreed with the simulation result.

**Conclusions: **In general, the new estimator performed more efficiently than the MLE, PRE, PLE and the PKL when multicollinearity was present.

## Introduction

A special case of the Generalized Linear Models (GLM) is the Poisson Regression Model (PRM) which is generally applied for count or frequency data modelling. Other count data models include: Bell regression model, Negative binomial regression model, zero inflated bell regression model, zero inflated regression model ( Amin *et al.*, 2020, 2021; Sami *et al.*, 2021; Rashad and Algamal, 2019; Majid *et al.*, 2021). The PRM is employed to model the relationship between a response variable and one or more explanatory variable where the response variable denotes a rare event or count data. The response variable also takes the form of a non-negative variable, and it is applicable in the following fields: economics, health, social and physical sciences. The Maximum Likelihood Estimation (MLE) method is popularly used to estimate the regression coefficient in a PRM. In both a Linear Regression Model (LRM) and Generalized Linear Model (GLM), MLE suffers a setback when the explanatory variables are correlated, which implies multicollinearity. Multicollinearity effects include large variance and regression coefficient covariances, negligible t-ratio and a high coefficient of determination (R-square) values. Alternative estimators to the MLE in the linear regression model include the ridge regression estimator by Hoerl and Kennard (1970), Liu estimator by Liu (1993), Liu-type estimator by Liu (2003), two-parameter estimator by Özkale and Kaciranlar (2007), *r*- *d* class estimator Kaçiranlar and Sakallioǧlu (2007), *k*- *d* class estimator Sakallioglu and Kaciranlar (2008), a two-parameter estimator by Yang and Chang (2010), modified two-parameter estimator by Dorugade (2014), modified ridge-type estimator by Lukman *et al*. (2019), modified Liu estimator by Lukman *et al*. (2020), Kibria-Lukman (KL) estimator by Kibria and Lukman (2020), modified new two-parameter estimator by Ahmad and Aslam (2020), the modified Liu ridge type estimator by Aslam and Ahmad (2020) and the DK estimator by Dawoud and Kibria (2020) among others. Researchers have extended some of these existing estimators in LRM to the PRM. Mansson *et al*. (2012) introduced the Liu estimator into the PRM. The modified **jackknifed ridge estimator** for the PRM was introduced by Türkan and Özel (2016). The ridge estimator was introduced into the PRM by Månsson and Shukur (2011). A new two-parameter for PRM was developed by Asar and Genç (2017). Recently, Poisson KL estimator was developed by Lukman *et al*. (2021) for combating multicollinearity in the PRM.

In this study, we propose the Modified Kibria-Lukman estimator to handle multicollinearity in PRM. The estimator is a single parameter estimator which makes it less computationally intensive as compared with the two-parameter estimators. Also, since the Kibria-Lukman estimator is found to outperform the Ridge and the Liu estimators, it is expected that the modification in this study will enhance the performance of the Kibria-Lukman estimator. Furthermore, we compared the performance of the estimator with the Poisson Maximum Likelihood Estimator (PMLE), Poisson Ridge Regression Estimator (PRE), Poisson Liu Estimator (PLE) and the Poisson KL estimator (PKLE).

## Methods

Given that the response variable, *y _i_* is in the form of count data, then it is assumed to follow a Poisson distribution as P _o_ ( *μ _i_*) where *μ _i_* = *e* ^( *x*_*i*_ *β*)^, and In *µ*_*i*_ = ( *x*_*i*_ *β*), *x _i_* is the *i* ^th^ row of matrix *X* which is a *n*×( *p*+ *1*) data matrix with *p* explanatory variables and *β* is a ( *p*+ *1*)× *1* vector of coefficients. The log likelihood of the model is given as:

$$l\left(\mu ;y\right)=\sum_{i=1}^{n}\gamma_{i}\log(e^{\left(xi\beta \right)})-{\left(\sum_{i=1}^{n}e^{\left(xi\beta \right)}\right)}_{i}\text{-}\log\left(\prod_{i=1}^{n}y_{i}!\right)$$ (2.1)

The most common method of maximizing the likelihood function is to use the iterated weighted least squares (IWLS) algorithm which results to:

$${\hat{\beta }}_{\mathrm{MLE}}={\left(X^{\prime }\hat{L}X\right)}^{-1}\left(X^{\prime }\hat{L}\hat{z}\right)$$ (2.2)

where $\hat{L}=\mathrm{diag}\left[{\hat{\mu }}_{i}\right]$ and $\hat{z}$ is a vector while the *i* ^th^ element equals ${\hat{z}}_{i}=\log({\hat{\mu }}_{i})+\frac{y_{i}-{\hat{\mu }}_{i}}{{\hat{\mu }}_{i}}.$

The MLE is normally distributed with a covariance matrix that is equivalent to the inverse of the second derivative as:

$$\mathrm{Cov}\left({\hat{\beta }}_{\mathrm{MLE}}\right)={\left(-E\left(\frac{\partial^{2}l}{\partial \beta_{j}\partial \beta \prime_{k}}\right)\right)}^{-1}={\left(X^{\prime }\hat{L}X\right)}^{-1}$$ (2.3)

and the mean squared error is given as:

$$E\left({\hat{\beta }}_{\mathrm{MLE}}\right)=E{\left({\hat{\beta }}_{\mathrm{MLE}}-\beta \right)}^{\prime }\left({\hat{\beta }}_{\mathrm{MLE}}-\beta \right)=\mathrm{tr}{\left(X^{\prime }\hat{L}X\right)}^{-1}=\sum_{j=1}^{P}\frac{1}{\lambda_{j}}$$ (2.4)

where $\lambda_{j}$ is the *j* ^th^ eigen value of the $\left(X^{\prime }\hat{V}X\right)$ matrix.

The Ridge estimator was adopted by Månsson and Shukur (2011) to solve multicollinearity problem in count data. The estimator is defined as follows:

$${\hat{\beta }}_{\mathrm{PRE}}={\left(X^{\prime }\hat{L}X+\mathrm{kI}\right)}^{-1}X^{\prime }\hat{L}X{\hat{\beta }}_{\mathrm{MLE}}$$ (2.5)

where $k=\left(\frac{1}{max(\alpha_{i}^{2})}\right)$ and (k>0).

The mean squared error is:

$$\mathrm{MSE}\left({\hat{\beta }}_{\mathrm{PRE}}\right)=\sum_{j=1}^{p}\frac{\lambda_{j}}{{\left(\lambda_{j}+k\right)}^{2}}+k^{2}\sum_{j=1}^{p}\frac{{\hat{\alpha }}_{j}^{2}}{{\left(\lambda_{j}+k\right)}^{2}}$$ (2.6)

*β*_*PRE*_ is effective in practice but it is a complicated function of the biasing parameter *k* ( Liu, 1993).

Mansson *et al*. (2012) developed the Liu estimator to the Poisson regression model as:

$${\hat{\beta }}_{\mathrm{PLE}}={\left(X^{\prime }\hat{L}X+I\right)}^{-1}\left(X\prime \hat{L}X+d\hat{\beta }\right){\hat{\beta }}_{\mathrm{ML}}$$ (2.7)

where

$$\hat{d}=\max\left(0,\frac{{\hat{\alpha }}_{j}^{2}-1}{{\hat{\alpha }}_{j}^{2}+\frac{1}{\lambda_{j}}}\right),0\leq d\leq 1.$$ (2.8)

The MSE for the Liu estimator is defined as:

$$\mathrm{MSE}\left({\hat{\beta }}_{\mathrm{PLE}}\right)=\sum_{j=1}^{P}\frac{{\left(\lambda_{j}+d\right)}^{2}}{\lambda_{j}{\left(\lambda_{j}+1\right)}^{2}}+{\left(d-1\right)}^{2}\sum_{j-1}^{p}\frac{\alpha_{j}^{2}}{{\left(\lambda_{j}+1\right)}^{2}}$$
_(2.9)_

where $\lambda_{j}$ is the *j* ^th^ eigenvalue of $X^{\prime }\hat{L}X$ and *α _j_* is the *j* ^th^ element of *α.*

The KL estimator was proposed by Kibria and Lukman (2020) as a means of mitigating the effect of multicollinearity on parameter estimation. The estimator is defined as

$${\hat{\beta }}_{\mathrm{KL}}={\left(X^{\prime }X+k\right)}^{-1}\left(X^{\prime }X-k\right){\hat{\beta }}_{\mathrm{MLE}}$$ (2.10)

By means of extension, the Poisson K-L estimator was proposed by Lukman *et al*. (2021) as follows:

$${\hat{\beta }}_{\mathrm{PKL}}={\left(X^{\prime }\hat{L}X+k\right)}^{-1}\left(X^{\prime }\hat{L}X-k\right){\hat{\beta }}_{\mathrm{MLE}}$$ (2.11)

$$\mathrm{MSE}\left({\hat{\beta }}_{\mathrm{PKL}}\right)=\sum_{j=1}^{p}\left(\frac{{\left(\lambda_{j}-k\right)}^{2}}{\lambda_{j}{\left(\lambda_{j}+k\right)}^{2}}\right)+4k^{2}\sum_{j=1}^{p}\left(\frac{\alpha_{j}^{2}}{{\left(\lambda_{j}+k\right)}^{2}}\right)$$ (2.12)

where $k=min\left(\frac{\alpha_{j}^{2}}{2\alpha_{j}^{2}+\frac{1}{\lambda_{j}}}\right)$ and k>0.

### The Poisson Modified KL estimator (PMKL)

The proposed estimator is obtained as follows: ${\hat{\beta }}_{\mathrm{MLE}}$ in equation (2.11) is replaced with the ridge estimator. Thus, we have:

$${\hat{\beta }}_{\mathrm{MKL}}={\left(X^{\prime }X+k\right)}^{-1}\left(X^{\prime }X-k\right){\left(X^{\prime }X+k\right)}^{-1}X^{\prime }y$$ (2.13)

The properties of the new estimator include:

$$E\left({\hat{\beta }}_{\mathrm{MKL}}\right)={\left(X^{\prime }X+\mathrm{kI}\right)}^{-1}\left(X^{\prime }X-\mathrm{kI}\right){\left(X^{\prime }X+\mathrm{kI}\right)}^{-1}X^{\prime }\mathrm{X\beta}$$ (2.14)

$$\text{Bias}\left({\hat{\beta }}_{\mathrm{MKL}}\right)={\left(X^{\prime }X+\mathrm{kI}\right)}^{-1}\left(X^{\prime }X-\mathrm{kI}\right){\left(X^{\prime }X+\mathrm{kI}\right)}^{-1}X^{\prime }\mathrm{X\beta}-\beta ={\left(X^{\prime }X+\mathrm{kI}\right)}^{-2}k\left[-3X^{\prime }X-\mathrm{kI}\right]\beta$$ (2.15)

The bias can be written in scalar form as:

$$\text{Bias}\left({\hat{\beta }}_{\mathrm{MKL}}\right)=k\sum_{j=1}^{p}\frac{\left(-3\lambda_{j}-k\right)\beta }{{\left(\lambda_{j}+k\right)}^{2}}$$ (2.16)

$$V\left({\hat{\beta }}_{\mathrm{MKL}}\right)=\sigma^{2}{\left(X^{\prime }X+\mathrm{kI}\right)}^{-1}\left(X^{\prime }X-\mathrm{kI}\right){\left(X^{\prime }X+\mathrm{kI}\right)}^{-1}X^{\prime }X{\left(X^{\prime }X+\mathrm{kI}\right)}^{-1}\left(X^{\prime }X-\mathrm{kI}\right){\left(X^{\prime }X+\mathrm{kI}\right)}^{-1}$$ (2.17)

$V\left({\hat{\beta }}_{\mathrm{MKL}}\right)$ can be represented in scalar form as follows:

$$V\left({\hat{\beta }}_{\mathrm{MKL}}\right)=\sum_{j=1}^{p}\frac{\lambda_{j}{\left(\lambda_{j}-k\right)}^{2}}{{\left(\lambda_{j}+k\right)}^{4}}$$ (2.18)

Thus, the MSE is obtained as:

$$\mathrm{MSE}\left({\hat{\beta }}_{\mathrm{MKL}}\right)=\sigma^{2}\sum_{j=1}^{p}\frac{\lambda_{j}{\left(\lambda_{j}-k\right)}^{2}}{{\left(\lambda_{j}+k\right)}^{4}}+k^{2}\sum_{j=1}^{p}\frac{{\left(3\lambda_{j}+k\right)}^{2}\beta^{2}}{{\left(\lambda_{j}+k\right)}^{4}}$$ (2.19)

The proposed estimator in (2.14) is extended to the PRM. It is referred to as the Poisson modified KL (PMKL) estimator and defined as:

$${\hat{\beta }}_{\text{PMKL}}={\left(X\prime \hat{L}X+k\right)}^{-1}\left(X^{\prime }\hat{L}X-k\right){\left(X^{\prime }\hat{L}X+k\right)}^{-1}X^{\prime }\hat{L}X{\hat{\beta }}_{\mathrm{MLE}}$$ (2.20)

The mean squared error of the PMKL is defined as:

$$\mathrm{MSE}\left({\hat{\beta }}_{\text{PMKL}}\right)=\sum_{j=1}^{p}\frac{\lambda_{j}{\left(\lambda_{j}-k\right)}^{2}}{{\left(\lambda_{j}+k\right)}^{4}}+k^{2}\sum_{j=1}^{p}\frac{{\left(3\lambda_{j}+k\right)}^{2}{\alpha_{j}}^{2}}{{\left(\lambda_{j}+k\right)}^{4}}$$ (2.21)

where $k=min\left[\frac{\sqrt{(3\lambda_{i}\alpha^{2}+\sigma^{2})24\alpha^{2}\sigma^{2}\lambda_{i}-3\lambda_{i}a^{2}+\sigma^{2}}}{2\beta^{2}}-(3\lambda_{i}\alpha^{2}+\sigma^{2})\right]$ and *k* > 0.

Suppose α=Q′β and $Q\prime X\prime \hat{L}XQ=\Lambda =\mathrm{diag}(\lambda_{1},\lambda_{2},..,\lambda_{p}).$ Where $\lambda_{1}\geq \lambda_{2},...,\geq \lambda_{p},$ Λ is the matrix of eigen-values of $X\prime \hat{L}X$ and *Q* is the matrix whose columns are the eigenvectors of $X\prime \hat{L}X$ .

The mean squared error (MSEM) and the following lemmas are adopted for theoretical comparisons among the estimators.

**Lemma 2.1** Let *A* be a positive definite (pd) matrix, that is, *A* > 0, and *a* be some vector, then $A-aa^{\prime }\geq 0$ if and only if (iff) $a\prime A^{-1}a\leq 1$ ( Farebrother, 1976).

**Lemma 2.2** $MSEM{\hat{\beta }}_{1}-MSEM{\hat{\beta }}_{2}=\delta^{2}D+b_{1}b\prime_{1}-b_{2}b\prime_{2}>0$ , if and only if ${b^{\prime }}_{2}{\left[\sigma^{2}D+b_{1}{b^{\prime }}_{1}\right]}^{-1}b_{2}<1$ where $MSE({\hat{\beta }}_{j})=V({\hat{\beta }}_{j})+b\prime_{j}b_{j},b_{1}=bias({\hat{\beta }}_{1})$ and $b_{2}=bias({\hat{\beta }}_{2})$ (Trenker and Toutenburg, 1990).

**Theorem 2.1:** ${\hat{\alpha }}_{PMKL}$ is preferred to ${\hat{\alpha }}_{PMLE}$ iff, $\text{MSEM}\left({\hat{\alpha }}_{PMLE}\right)-\text{MSEM}\left({\hat{\alpha }}_{PMKL}\right)>0$ provided *k* > 0.

*Proof*

$$V\left({\hat{\alpha }}_{\text{PMLE}}\right)-V\left({\hat{\alpha }}_{\text{PMKL}}\right)=\text{Qdiag}{\left\{\frac{1}{\lambda_{j}}-\frac{\lambda_{j}{\left(\lambda_{j}-k\right)}^{2}}{{\left(\lambda_{j}+k\right)}^{4}}\right\}}_{j=1}^{P}Q^{\prime }$$

It is observed that ${\left(\lambda_{j}+k\right)}^{4}-\lambda_{j}^{2}{\left(\lambda_{j}-k\right)}^{2}>0$ such that the expression above is non-negative for k > 0

**Theorem 2.2:** ${\hat{\alpha }}_{PMKL}$ is preferred to ${\hat{\alpha }}_{PRE}$ iff, $\text{MSEM}\left({\hat{\alpha }}_{PRE}\right)-\text{MSEM}\left({\hat{\alpha }}_{PMKL}\right)>0$ provided *k* > 0.

*Proof*

$$V\left({\hat{\alpha }}_{PRE}\right)-V\left({\hat{\alpha }}_{PMKL}\right)=\text{Qdiag}{\left\{\frac{\lambda_{j}}{{\left(\lambda_{j}+k\right)}^{2}}-\frac{\lambda_{j}{\left(\lambda_{j}-k\right)}^{2}}{{\left(\lambda_{j}+k\right)}^{4}}\right\}}_{j=1}^{p}Q^{\prime }$$

We can observe that the difference of the variance of the estimator is non-negative since ${\left(\lambda_{j}+k\right)}^{2}-\left({\lambda_{j}}^{2}-k^{2}\right)>0$ for *k* > 0.

**Theorem 2.3:** ${\hat{\alpha }}_{PMKL}$ is preferred to ${\hat{\alpha }}_{PLE}$ iff, $\text{MSEM}\left({\hat{\alpha }}_{PLE}\right)-\text{MSEM}\left({\hat{\alpha }}_{PMKL}\right)>0$ provided *k* > 0 and 0 < *d* < 1.

*Proof*

$$V\left({\hat{\alpha }}_{PLE}\right)-\mathrm{Cov}\left({\hat{\alpha }}_{PMKL}\right)=\text{Qdiag}{\left\{\frac{{\left(\lambda_{j}+d\right)}^{2}}{\lambda_{j}{\left(\lambda_{j}+1\right)}^{2}}-\frac{\lambda_{j}{\left(\lambda_{j}-k\right)}^{2}}{{\left(\lambda_{j}+k\right)}^{4}}\right\}}_{j=1}^{p}Q^{\prime }$$

The difference of the variance is non-negative since

$\left(\lambda_{j}+k\right)\left(\lambda_{j}+d\right)-\lambda_{j}\left(\lambda_{j}+1\right)\left(\lambda_{j}-k\right)>0$ for 0 < *d* < 1 and *k* > 0.

**Theorem 2.4:** ${\hat{\alpha }}_{PMKL}$ is preferred to ${\hat{\alpha }}_{PKL}$ iff, $\text{MSEM}\left({\hat{\alpha }}_{PKL}\right)-\text{MSEM}\left({\hat{\alpha }}_{PMKL}\right)>0$ provided *k* > 0.

*Proof*

$$V\left({\hat{\alpha }}_{PKL}\right)-\mathrm{Cov}\left({\hat{\alpha }}_{PMKL}\right)=\text{Qdiag}{\left\{\frac{{\left(\lambda_{j}-k\right)}^{2}}{\lambda_{j}{\left(\lambda_{j}+k\right)}^{2}}-\frac{\lambda_{j}{\left(\lambda_{j}-k\right)}^{2}}{{\left(\lambda_{j}+k\right)}^{4}}\right\}}_{j=1}^{p}Q^{\prime }$$

The difference of the variance is non-negative since $\left(\lambda_{j}+k\right)\left(\lambda_{j}-k\right)-\lambda_{j}\left(\lambda_{j}-k\right)>0$ for *k* > 0.

### Selection of biasing parameter

The biasing parameter *k* for the estimator is obtained by differentiating the MSE in equation (2.21) with respect to *k* as follows:

$$\partial MSE({\hat{\beta }}_{MKL})=-\left[2\sigma^{2}\sum_{j=1}^{p}\frac{\lambda_{j}(\lambda_{j}-k)}{(\lambda_{j}+k{)}^{4}}+4\sigma^{2}\sum_{j=1}^{p}\frac{\lambda_{j}(\lambda_{j}-k{)}^{2}}{(\lambda_{j}+k{)}^{5}}\right]+\left[2k\beta^{2}\sum_{j=1}^{p}\frac{\left(3\lambda_{j}+k)[(3\lambda_{j}+k)+K\right]}{(\lambda_{j}+k{)}^{4}}-4k^{2}\sum_{j=1}^{p}\frac{(3\lambda_{j}+k{)}^{2}\beta^{2}}{(\lambda_{j}+k{)}^{5}}\right]$$ (2.22)

By equating to 0 and dividing through by 2 we have the resulting equation as:

$$-\sum_{j=1}^{p}\left[\sigma^{2}\frac{\left(\lambda_{j}(\lambda_{j}-k\right)}{(\lambda_{j}+k{)}^{4}}+2\sigma^{2}\frac{\lambda_{j}(\lambda_{j}-k{)}^{2}}{(\lambda_{j}+k{)}^{5}}\right]+\sum_{j=1}^{p}\left[k\beta^{2}\frac{\left(3\lambda_{j}+k)(3\lambda_{j}+2k\right)}{(\lambda_{j}+k{)}^{4}}-2k^{2}\frac{(3\lambda_{j}+k{)}^{2}\beta^{2}}{(\lambda_{j}+k{)}^{5}}\right]=0$$ (2.23)

$$-\sigma^{2}\lambda_{j}(\lambda_{j}-k)[\lambda_{j}+k+2\lambda_{j}-2k)]=k(3\lambda_{j}+k)\beta^{2}[6k\lambda_{j}+2k^{2}-3\lambda_{j}^{2}-5k\lambda_{j}-2k^{2}]-\sigma^{2}\lambda_{j}(\lambda_{j}-k)[3\lambda_{j}-k)]=k(3\lambda_{j}+k)\beta^{2}[k\lambda_{j}-3\lambda_{j}^{2}]$$ (2.24)

Solving the equation above for *k* yields the biasing parameter *k* given below as:

$$k_{\mathrm{MKL}}=\min\left[\frac{\sqrt{{\left(3\lambda_{i}\alpha^{2}+\sigma^{2}\right)}^{2}+4\alpha^{2}\sigma^{2}\lambda_{i}-3\lambda_{i}\alpha^{2}+\sigma^{2}}}{2\beta^{2}}-\left(3\lambda_{i}\alpha^{2}+\sigma^{2}\right)\right]$$ (2.25)

The shrinkage parameter estimated by Mansson and Shukur, (2011) and Kibria and Lukman (2020) was also adopted for this study as listed:

$$k_{1}=\frac{1}{\max\left(\alpha_{j}^{2}\right)}$$ (2.26)

$$k_{2}=\frac{p}{\sum \left(2\alpha_{j}^{2}+\frac{1}{\lambda_{j}e}\right)}$$ (2.27)

$$k_{3}=k_{MKL}=k_{3}=\min\left[\frac{\sqrt{(3\lambda_{j}\alpha^{2}+\sigma^{2}{)}^{2}+4\alpha^{2}\sigma^{2}\lambda_{j}-3\lambda_{j}\alpha^{2}+\alpha^{2}}}{2\beta^{2}}-(3\lambda_{j}\alpha^{2}+\sigma^{2})\right]$$ (2.28)

*k*_1_ and *k*_2_ is the biasing parameter for PMKL1 and PMKL2, while *k*_3_ is the biasing parameters for PMKL3.

## Simulation Design and Real-Life Application

### Simulation study and result

In this section, a simulation study is carried out to compare the performance of the different estimators. The generation of the dependent variables are done using pseudo-random numbers from P _o_ ( *μ _i_*) where $\mu_{i}=e^{x_{i}\beta }i=1,2,\ldots ,n$ and *X*_*i*_ is the *i* ^th^ row of the design matrix with $\beta =\left(\beta_{0},\beta_{1},\ldots ,\beta_{p}\right)$ being the coefficient vector. The generation of the explanatory variables with different levels of correlation is obtained using

xij=1−ρ212zij+ρzip+1;i=1,2,...,nandj=1,2,...,p. (3.1)

where ρ is the level of multicollinearity between the explanatory variables ( Kibria *et al.* 2015; Kibria and Banik, 2016; Lukman *et al*., 2019b, Lukman *et al*. 2020b). $z_{\mathrm{ij}}$ are pseudo-random numbers generated using the standard normal distribution such that *i* ranges from *1* to *n* and *j* from *1* to *p.* As a common restriction used in simulation studies, it is assumed that $\sum_{j=1}^{p}\beta_{j}^{2}=1$ and $\beta_{1}=\beta_{2}=\ldots =\beta_{p}.$ Also, the effect of the intercept value is also being investigated as values are taken to be 1, 0 and -1 (Kibria *et al*. 2014). The different levels of correlation taken are 0.8, 0.9, 0.95, 0.99 and 0.999. The other factors varied in the simulation study are the sample size *n* and the number of explanatory variable *p.* We assume *n* = 50, 100 and 200 observations and *p* = 4 and 8 explanatory variables.

The simulation results in Tables 1 to 6 that for each of the estimators, the simulated MSE values increase as the multicollinearity level increases, keeping other factors constant. There is also an increase in the mean squared error as the sample size increases for all estimators compared while other factors were kept constant. As the intercept values varied from -1 to +1, the values of the mean squared error reduced for all estimators. Result shows that the PMKL1 performed best with minimum MSE at varying sample sizes. It was closely followed by PMKL2. They are both considered more suitable for estimation of parameters in the Poisson regression model than the MLE as it performed worst when multicollinearity is a challenge. In general, the PMKL1 estimator consistently performed more efficiently than the MLE, PRE, PLE and the PKL estimators.

**Table 1.** Simulation result for mean squared error (MSE) when P = 4 and intercept = 1.

$\beta_{0}$	N	ρ	MLE	PRE	PLE	PKL	PMKL1	PMKL2	PMKL3
1	50	0.8	0.0389	0.0376	0.0384	0.0383	0.0366	0.0366	0.0383
		0.9	0.0534	0.0494	0.0520	0.0515	0.0440	0.0446	0.0515
		0.95	0.0852	0.0729	0.0808	0.0791	0.0553	0.0574	0.0806
		0.99	0.3548	0.2013	0.2800	0.2435	0.0696	0.0750	0.2835
		0.999	3.4244	0.8757	1.8191	0.2302	0.1187	0.1041	1.4952
	100	0.8	0.0107	0.0108	0.0107	0.0107	0.0113	0.0111	0.0107
		0.9	0.0125	0.0124	0.0125	0.0125	0.0125	0.0125	0.0125
		0.95	0.0182	0.0177	0.0181	0.0181	0.0171	0.0172	0.0181
		0.99	0.0691	0.0595	0.0670	0.0665	0.0451	0.0480	0.0682
		0.999	0.6465	0.2995	0.5061	0.4404	0.0852	0.0979	0.6098
	200	0.8	0.0057	0.0056	0.0056	0.0056	0.0060	0.0059	0.0056
		0.9	0.0068	0.0068	0.0068	0.0068	0.0067	0.0068	0.0067
		0.95	0.0105	0.0104	0.0105	0.0105	0.0103	0.0104	0.0105
		0.99	0.0422	0.0394	0.0416	0.0415	0.0347	0.0357	0.0419
		0.999	0.5234	0.1897	0.4355	0.2322	0.0419	0.0324	0.5211

MLE = Maximum Likelihood Estimator; PRE=Poisson Ridge Estimator; PLE = Poisson Liu Estimator; PKL = Poisson Kibria-Lukman Estimator; PMKL1 = Poisson Modified Kibria Lukman Estimator 1; PMKL2 = Poisson Modified Kibria Lukman Estimator 2; PMKL3 = Poisson Modified Kibria Lukman Estimator 3.

**Table 2.** Simulation result for mean squared error (MSE) when P = 4 and intercept = 0.

$\beta_{0}$	n	ρ	MLE	PRE	PLE	PKL	PMKL1	PMKL2	PMKL3
0	50	0.8	0.1091	0.0821	0.1003	0.1000	0.0474	0.0532	0.1036
		0.9	0.1479	0.0978	0.1303	0.1295	0.0505	0.0556	0.1386
		0.95	0.2356	0.1287	0.1910	0.1866	0.0536	0.0588	0.2158
		0.99	0.9393	0.2847	0.5410	0.3425	0.0565	0.0593	0.6883
		0.999	9.4757	1.9349	4.8030	2.2184	0.2562	0.1817	2.9303
	100	0.8	0.0295	0.0266	0.0291	0.0291	0.0230	0.0238	0.0294
		0.9	0.0340	0.0301	0.0335	0.0335	0.0243	0.0258	0.0339
		0.95	0.0500	0.0416	0.0488	0.0488	0.0297	0.0325	0.0497
		0.99	0.1896	0.1088	0.1712	0.1706	0.0420	0.0503	0.1867
		0.999	3.5624	1.1897	1.5432	0.7168	0.0945	0.0991	1.5706
	200	0.8	0.0154	0.0153	0.0153	0.0153	0.0138	0.0139	0.0154
		0.9	0.0178	0.0187	0.0187	0.0187	0.0161	0.0166	0.0188
		0.95	0.0262	0.0284	0.0284	0.0284	0.0223	0.0234	0.0286
		0.99	0.8292	0.1083	0.1083	0.1082	0.0454	0.0529	0.1126
		0.999	1.5185	0.3222	0.8548	0.1183	0.0543	0.0743	0.9527

MLE = Maximum Likelihood Estimator; PRE = Poisson Ridge Estimator; PLE = Poisson Liu Estimator; PKL = Poisson Kibria-Lukman Estimator; PMKL1 = Poisson Modified Kibria Lukman Estimator 1; PMKL2 = Poisson Modified Kibria Lukman Estimator 2; PMKL3 = Poisson Modified Kibria Lukman Estimator 3.

**Table 3.** Simulation result for mean squared error (MSE) when P = 4 and intercept = -1.

$\beta_{0}$	n	ρ	MLE	PRE	PLE	PKL	PMKL1	PMKL2	PMKL3
-1	50	0.8	0.3089	0.2230	0.2569	0.2211	0.2093	0.2146	0.2478
		0.9	0.4295	0.2604	0.3327	0.2702	0.2299	0.2366	0.3205
		0.95	0.6924	0.3372	0.4890	0.3364	0.2562	0.2755	0.4573
		0.99	2.7802	0.7723	1.5114	0.3302	0.3020	0.3734	1.0038
		0.999	26.9726	5.4928	14.1059	9.2016	0.7486	1.9380	4.1156
	100	0.8	0.0809	0.0772	0.0775	0.0764	0.0533	0.0739	0.0778
		0.9	0.0935	0.0834	0.0891	0.0886	0.1043	0.1004	0.0812
		0.95	0.1389	0.1116	0.1290	0.1287	0.1175	0.1099	0.1296
		0.99	0.5161	0.2666	0.4024	0.3878	0.1281	0.1231	0.4790
		0.999	4.6805	1.1257	2.6775	0.7517	0.1994	0.2043	3.5989
	200	0.8	0.0421	0.0426	0.0417	0.0409	0.0404	0.0423	0.0409
		0.9	0.0511	0.0498	0.0501	0.0498	0.0446	0.0535	0.0499
		0.95	0.0767	0.0704	0.0741	0.0741	0.0672	0.0633	0.0741
		0.99	0.3107	0.2115	0.2766	0.2731	0.1226	0.1309	0.3016
		0.999	4.1247	0.8450	2.0275	0.7102	0.1369	0.1528	2.6541

MLE = Maximum Likelihood Estimator; PRE = Poisson Ridge Estimator; PLE = Poisson Liu Estimator; PKL = Poisson Kibria-Lukman Estimator; PMKL1 = Poisson Modified Kibria Lukman Estimator 1; PMKL2 = Poisson Modified Kibria Lukman Estimator 2; PMKL3 = Poisson Modified Kibria Lukman Estimator 3.

**Table 4.** Simulation result for mean squared error (MSE) when P = 8 and intercept = 1.

$\beta_{0}$	n	ρ	MLE	PRE	PLE	PKL	PMKL1	PMKL2	PMKL3
1	50	0.8	0.0980	0.0883	0.0960	0.0951	0.0796	0.0804	0.0969
		0.9	0.1404	0.1163	0.1355	0.1329	0.0906	0.0938	0.1369
		0.95	0.2256	0.1631	0.2113	0.2036	0.1004	0.1072	0.2201
		0.99	0.9255	0.3956	0.7190	0.5837	0.1133	0.1256	0.8598
		0.999	8.4713	1.9414	4.8816	1.0453	0.3070	0.2788	5.8787
	100	0.8	0.0232	0.0229	0.0231	0.0231	0.0227	0.0227	0.0231
		0.9	0.0340	0.0329	0.0337	0.0335	0.0314	0.0316	0.0336
		0.95	0.0534	0.0499	0.0525	0.0518	0.0440	0.0449	0.0526
		0.99	0.2226	0.1634	0.2026	0.1891	0.0853	0.0792	0.2081
		0.999	2.1185	0.7284	1.2950	0.4499	0.0882	0.0951	1.2901
	200	0.8	0.0057	0.0057	0.0057	0.0057	0.0056	0.0057	0.0057
		0.9	0.0076	0.0076	0.0076	0.0076	0.0075	0.0074	0.0076
		0.95	0.0117	0.0115	0.0116	0.0116	0.01130	0.01134	0.0116
		0.99	0.0443	0.0412	0.0434	0.0430	0.0356	0.0365	0.0436
		0.999	1.8722	0.2071	0.5491	0.2671	0.0566	0.0562	0.8730

MLE = Maximum Likelihood Estimator; PRE = Poisson Ridge Estimator; PLE=Poisson Liu Estimator; PKL = Poisson Kibria-Lukman Estimator; PMKL1 = Poisson Modified Kibria Lukman Estimator 1; PMKL2 = Poisson Modified Kibria Lukman Estimator 2; PMKL3 = Poisson Modified Kibria Lukman Estimator 3.

**Table 5.** Simulation result for mean squared error (MSE) when P = 8 and intercept = 0.

$\beta_{0}$	n	ρ	MLE	PRE	PLE	PKL	PMKL1	PMKL2	PMKL3
0	50	0.8	0.2738	0.1473	0.2377	0.2352	0.0808	0.0879	0.2682
		0.9	0.3927	0.1834	0.3258	0.3201	0.0829	0.0899	0.3825
		0.95	0.6114	0.2382	0.4677	0.4448	0.0942	0.0952	0.5888
		0.99	2.4858	0.6218	1.4882	0.7865	0.1205	0.1428	2.1778
		0.999	23.3548	4.6860	13.2573	6.5291	0.7055	0.4636	12.7807
	100	0.8	0.0646	0.0554	0.0617	0.0617	0.0491	0.0491	0.0635
		0.9	0.0934	0.0750	0.0879	0.0878	0.0526	0.0578	0.0907
		0.95	0.1462	0.1041	0.1327	0.1324	0.0553	0.0593	0.1395
		0.99	0.6068	0.2589	0.4234	0.2577	0.0587	0.0643	0.5154
		0.999	5.6854	1.4178	2.9951	0.3878	0.1433	0.1070	2.2332
	200	0.8	0.0159	0.0151	0.0157	0.0157	0.0141	0.0143	0.0158
		0.9	0.0207	0.0196	0.0205	0.0205	0.0176	0.0181	0.0206
		0.95	0.0319	0.0290	0.0312	0.0312	0.0242	0.0254	0.0315
		0.99	0.1185	0.0857	0.1089	0.1087	0.0441	0.0506	0.1141
		0.999	1.7596	0.4612	1.0889	0.1810	0.0794	0.0921	1.0001

MLE = Maximum Likelihood Estimator; PRE = Poisson Ridge Estimator; PLE = Poisson Liu Estimator; PKL=Poisson Kibria-Lukman Estimator; PMKL1 = Poisson Modified Kibria Lukman Estimator 1; PMKL2 = Poisson Modified Kibria Lukman Estimator 2; PMKL3 = Poisson Modified Kibria Lukman Estimator 3.

**Table 6.** Simulation result for mean squared error (MSE) when P = 8 and intercept = -1.

$\beta_{0}$	n	ρ	MLE	PRE	PLE	PKL	PMKL1	PMKL2	PMKL3
-1	50	0.8	0.8248	0.259	0.6469	0.4512	0.2159	0.6700	0.7314
		0.9	1.1355	0.4945	0.8253	0.5699	0.4693	0.5526	0.9927
		0.95	1.7701	0.6264	1.1745	0.6921	0.5848	0.5614	1.4256
		0.99	7.1964	1.6865	4.2093	1.0061	0.4735	0.4493	4.8258
		0.999	65.8760	12.8251	38.0726	37.7305	2.0409	0.9017	24.8129
	100	0.8	0.1800	0.1542	0.1653	0.1493	0.1422	0.1616	0.1547
		0.9	0.2575	0.2001	0.2269	0.2073	0.2020	0.1858	0.2131
		0.95	0.4105	0.2729	0.3392	0.3019	0.2354	0.2115	0.3419
		0.99	1.6914	0.6536	1.0983	0.5039	0.2420	0.2180	1.1012
		0.999	15.5667	3.7188	8.3620	4.0565	0.3710	0.2221	3.8870
	200	0.8	0.0436	0.0422	0.0425	0.0419	0.0303	0.0475	0.0419
		0.9	0.0568	0.0535	0.0546	0.0544	0.0512	0.0545	0.0542
		0.95	0.0860	0.0766	0.0810	0.0808	0.0693	0.0691	0.0813
		0.99	0.3260	0.2168	0.2753	0.2562	0.1022	0.1087	0.2890
		0.999	8.7594	1.5639	3.3266	2.0065	0.2433	0.1621	1.7855

MLE = Maximum Likelihood Estimator; PRE = Poisson Ridge Estimator; PLE = Poisson Liu Estimator; PKL = Poisson Kibria-Lukman Estimator; PMKL1 = Poisson Modified Kibria Lukman Estimator for k1; PMKL2 = Poisson Modified Kibria Lukman Estimator for k _2_; PMKL3 = Poisson Modified Kibria Lukman Estimator for k _3_.

## Real Life Application

Having carried out a simulation study, the efficacy of the proposed estimator needs to be further investigated by considering a real-life application. The Poisson regression model has been applied to the aircraft damage dataset initially by Myers *et al*. (2012) and subsequently by other researchers such as Asar and Genc (2017) and Amin *et al*. (2020) among others. By following the Pearson chi-square goodness of fit test, Amin *et al*. (2020) was able to ascertain that the data fits a Poisson regression model. The test confirms the suitability of the response variable to Poisson distribution with P-value of 6.898122 (0.07521). The dataset provides some detail on two separate aircrafts: The McDonnell Douglas A-4 Skyhawk and the A-6 Grumman Itruder. The dependent variable denotes the number of locations with damage on the aircraft and this follows a Poisson distribution (Asar and Genc, 2017; Amin *et al*., 2020). The data set has three explanatory variables, X _1_ shows the type of aircraft which makes the outcome binary (A-4 is coded as 0 and A-6 is coded as 1). X2 is the bomb load in tons and X3 is the number of months of aircrew experience. Meyers *et al*. (2012) was able to ascertain that the data set is greatly affected by multicollinearity. The eigenvalues of the matrix *X* were obtained as 4.3333, 374.8961 and 2085.2251. The condition number of 219.3654 was also obtained which is an indication of the problem of multicollinearity since it is greater than 30 (Asar and Genc, 2017). The performance of the estimators is judged based on the mean squared error of each of the estimators.

From Table 7, it is evident that all of the regression coefficients had identical signs. The estimator with the highest mean squared error is the MLE due to the presence of multicollinearity. The suggested estimator (PMKL1, PMKL2, PMKL3) has the lowest MSE that has established its dominance. We also observed that the performance of the estimator is highly dependent on the biasing parameter *k*. The expressions for the biasing parameters are defined in equation (2.26)- (2.28).

**Table 7.** Regression coefficients and MSE.

coef.	MLE	PRE	PLE	PKL	PMKL1 (k1)	PMKL2 (k2)	PMKL3 (k3)
${\hat{\alpha }}_{0}$	-0.406	-0.167	-0.255	-0.107	-0.019	-0.002	-0.077
${\hat{\alpha }}_{1}$	0.569	0.380	0.479	0.391	0.120	0.179	0.322
${\hat{\alpha }}_{2}$	0.165	0.171	0.167	0.168	0.183	0.179	0.172
${\hat{\alpha }}_{3}$	-0.014	-0.015	-0.015	-0.016	-0.017	-0.017	-0.016
MSE	1.029	0.273	0.432	0.225	0.083	0.095	0.092
*k*		2.5444	0.1120	1.9409	2.5444	1.9409	0.9905

MLE = Maximum Likelihood Estimator; PRE = Poisson Ridge Estimator; PLE = Poisson Liu Estimator; PKL = Poisson Kibria-Lukman Estimator; PMKL1 = Poisson Modified Kibria Lukman Estimator 1; PMKL2 = Poisson Modified Kibria Lukman Estimator 2; PMKL3 = Poisson Modified Kibria Lukman Estimator 3.

## Conclusion

The parameters in the PRM are commonly estimated using the Maximum Likelihood Estimator. However, literature had shown that the estimator suffers a setback when the explanatory variables are correlated. This problem led to the implementation of alternative estimators with single shrinkage parameters such as the Poisson Ridge Regression Estimator (PRE), Poisson Liu Estimator (PLE) and the Poisson KL Estimator (PKLE). The KL estimator was generally preferred to the ridge regression and Liu estimator in the linear regression model. According to Lukman *et al*. (2021), the Poisson KL estimator outperforms PRE and PLE. This study modified the KL estimator to propose a new estimator called the Poisson Modified KL estimator (PMKL). The new estimator falls in the same class with the ridge, Liu and KL estimators since they possessed a single shrinkage parameter. We investigated the performance of the estimators with a simulation study and a real-life application. From the results, we observed that the new estimator consistently performed well in the presence of multicollinearity with the lowest MSE. Finally, the new estimator is more suitable to combat multicollinearity in the PRM.

## Data availability

All data underlying the results are available as part of the article and no additional source data are required.
