# Supplementary material for: Modified Kibria-Lukman (MKL) estimator for the Poisson Regression Model: application and simulation
Source: F1000Res. 2021 Dec 14;10:548. Originally published 2021 Jul 8. [Version 2] doi: 10.12688/f1000research.53987.2 (PMC8825644; doi:10.12688/f1000research.53987.2)
Supplement: Peer review history

# Peer review: Reviewer response for version 2

**DOI:** 10.5256/f1000research.78664.r113239

**Published:** 2021-12-21

**Authors:** Muhammad Amin

**Affiliations:** 1 Department of Statistics, University of Sargodha, Sargodha, Pakistan

This paper can be accepted for indexing after incorporation of the following minor points:

1. Check equations 2.1 and 3.1.
2. Change "PMKL" to "PMKLE" in the whole manuscript.
3. In the application section, change "eigenvalues of the matrix X were" to "eigenvalues of the matrix X'X were".
4. In the application section, report the estimated values of the biasing parameters of the considered biased estimators with proper equation citations.
5. Change " Sami et al., 2021" to "Sami et al., 2022".

Is the work clearly and accurately presented and does it cite the current literature?

Partly

If applicable, is the statistical analysis and its interpretation appropriate?

Partly

Are all the source data underlying the results available to ensure full reproducibility?

Partly

Is the study design appropriate and is the work technically sound?

Yes

Are the conclusions drawn adequately supported by the results?

Yes

Are sufficient details of methods and analysis provided to allow replication by others?

Yes

Reviewer Expertise:

Regression Analysis, Biased Estimation Methods

I confirm that I have read this submission and believe that I have an appropriate level of expertise to confirm that it is of an acceptable scientific standard.

# Peer review: Reviewer response for version 2

**DOI:** 10.5256/f1000research.78664.r113241

**Published:** 2021-12-15

**Authors:** Mohammad Arashi

**Affiliations:** 1 Department of Statistics, Faculty of Mathematical Sciences, Ferdowsi University of Mashhad, Mashhad, Iran

The authors covered all the raised comments and I am happy with the current version.

Is the work clearly and accurately presented and does it cite the current literature?

Yes

If applicable, is the statistical analysis and its interpretation appropriate?

Yes

Are all the source data underlying the results available to ensure full reproducibility?

Partly

Is the study design appropriate and is the work technically sound?

Yes

Are the conclusions drawn adequately supported by the results?

Yes

Are sufficient details of methods and analysis provided to allow replication by others?

Yes

Reviewer Expertise:

High-dimensional modeling; shrinkage estimation

I confirm that I have read this submission and believe that I have an appropriate level of expertise to confirm that it is of an acceptable scientific standard.

# Peer review: Reviewer response for version 1

**DOI:** 10.5256/f1000research.57427.r89263

**Published:** 2021-08-09

**Authors:** Nimet Özbay

**Affiliations:** 1 Department of Statistics, Faculty of Science and Letters, Çukurova University, Adana, Turkey

This article focuses on proposing a modified KL estimator to mitigate the Poisson Regression Model with multicollinearity. Some theoretical properties of the new estimator are examined. A numerical example is conducted to show the performance of the new estimator. I think the authors should give the definition of the modified KL estimator in more detail and explain its statistical necessity. The organization of the paper, grammatical mistakes, and punctuation errors should also be controlled. This article may be indexed after the major comments below are applied.

**Comments:**

 The main document of the article does not contain line numbers, thus it has been quite difficult to pinpoint the location of the comments.

1. There are lots of language and punctuation errors throughout the whole article, so the authors should recheck the writing of the manuscript. For example:
   
  - In the Abstract, “the simulation result showed…” should be changed to “the simulation result showed that…”.
  - On page 3, a dot is required before equation (2.1).
  - On page 3, a comma is required before equation (2.2).
  - On page 5, line 1, “estimator” should be “estimators”.
  - Section number is required for the sections, etc.
  - The basic punctuation marks are missing throughout the paper.
2. On page 3, the abbreviation “PRE” is repeated.
3. In the whole article, there are some inappropriate uses of the abbreviations. The authors should rearrange the use of abbreviations. In some places, previously made abbreviations are repeated.
4. The use of “hat” is missing while presenting some estimators.
5. In the Introduction section, the manuscript *Defining a two-parameter estimator: a mathematical programming evidence *by Üstündağ Şiray *et al.* (2021) ^1^ may be mentioned since this is a more recent article in which a new biased estimator is proposed to mitigate multicollinearity.
6. On page 4, the authors should explain what lambdas are.
7. On page 6, before equation (2.8), “means square error” should be “MSE”.
8. There is no explanation for equation (2.11).
9. I think, “equation (2.10)” should be “equation (2.9)” before equation (2.12) on page 4.
10. On page 5, the authors should explain what lambdas are. Do the authors use “V” to Show variance? If so, some explanations should be added about it.
11. On page 5, there is the incorrect use of “MSEM”. This abbreviation does not exist, although it is used while representing the lemmas and theorems.
12. I think the authors employ the canonical form in the proof of the theorems. Unfortunately, I did not find some information about the canonical model.
13. The selection of the biasing parameter section is insufficient. A detailed derivation and more information should be given.
14. In the simulation section, on page 7, why does the mean square error increase as the sample size increases?
15. It would be better if no abbreviations were used in the title.

Is the work clearly and accurately presented and does it cite the current literature?

Partly

If applicable, is the statistical analysis and its interpretation appropriate?

Yes

Are all the source data underlying the results available to ensure full reproducibility?

Partly

Is the study design appropriate and is the work technically sound?

Yes

Are the conclusions drawn adequately supported by the results?

Partly

Are sufficient details of methods and analysis provided to allow replication by others?

Partly

Reviewer Expertise:

Monte Carlo simulation, Linear regression model, Econometric models, Applied statistics, Biased estimation

I confirm that I have read this submission and believe that I have an appropriate level of expertise to confirm that it is of an acceptable scientific standard, however I have significant reservations, as outlined above.

## References

[1] : Defining a two-parameter estimator: a mathematical programming evidence. *Journal of Statistical Computation and Simulation* .2021;91(11) : 10.1080/00949655.2021.1885671 2133-2152 10.1080/00949655.2021.1885671

# Peer review: Reviewer response for version 1

**DOI:** 10.5256/f1000research.57427.r89258

**Published:** 2021-08-03

**Authors:** Muhammad Amin

**Affiliations:** 1 Department of Statistics, University of Sargodha, Sargodha, Pakistan

In this paper, the authors introduced a new estimator by modified KL estimator for the Poisson regression model to overcome the effect of multicollinearity. The paper is original and deals with a topic of interest. This paper could be accepted for indexing after incorporating the following points.

  

- Write one paragraph on count data models and their importance at the start of the Introduction and include some citations that demonstrate the importance of count data models, for example: Amin *et al*., 2020 ^1^; Amin *et al*., 2021 ^2^; Sami *et al.,* 2021 ^3^; Amin *et al*., 2021 ^4^; Majid *et al*., 2021 ^5^; Rashad *et al*., 2019 ^6^; Algamal *et al*., 2015 ^7^; Algamal *et al*., 2021 ^8^; Alanaz *et al*., 2018 ^9^.
- Write the reason for your proposed estimator over other estimators in the last paragraph of the Introduction section.
- Change independent variables to explanatory variables in the whole study.
- Write the first paragraph clearly and correct equation 2.1 of the Methods section by following Amin *et al*., 2020 ^1^.
- Change “mean square error” to “mean squared error” in the whole manuscript.
- On page 3, write the reason for adapting the Poisson ridge estimator.
- On page 4, line 1, write the range of ridge parameter k.
- Write the limitations of the ridge estimator after equation (2.5).
- Write the range of Liu parameter d after equation (2.6).
- Write different notations of the ridge parameter k, for ridge, KL, and MKL estimators and also mention the ranges of these biasing parameters.
- Write the expressions for MSEs of ridge, Liu, and KL estimators.
- In Lemma 2.2, define b1, b2,
- The statement of Theorem 2.2 is wrong, I suggest the authors correct this.
- Define e in equation (2.23).
- In equation (2.24), change *λ* *i*   to *λ* *j*
- Correct expressions above equation (3.1).
- The interpretations of simulation results need more detailed discussion.
- In real application, report the estimated values of each biasing parameter with proper citation of equations. Moreover, cite equation to compute MSE of the consider estimators.
- There are some grammatical issues that should be corrected.

Is the work clearly and accurately presented and does it cite the current literature?

Partly

If applicable, is the statistical analysis and its interpretation appropriate?

Partly

Are all the source data underlying the results available to ensure full reproducibility?

Partly

Is the study design appropriate and is the work technically sound?

Yes

Are the conclusions drawn adequately supported by the results?

Yes

Are sufficient details of methods and analysis provided to allow replication by others?

Yes

Reviewer Expertise:

Regression Analysis, Biased Estimation Methods

I confirm that I have read this submission and believe that I have an appropriate level of expertise to confirm that it is of an acceptable scientific standard, however I have significant reservations, as outlined above.

## References

[1] : On the James-Stein estimator for the poisson regression model. *Communications in Statistics - Simulation and Computation* .2020; 10.1080/03610918.2020.1775851 1-13 10.1080/03610918.2020.1775851

[2] : On the estimation of Bell regression model using ridge estimator. *Communications in Statistics - Simulation and Computation* .2021; 10.1080/03610918.2020.1870694 1-14 10.1080/03610918.2020.1870694

[3] : On the ridge estimation of theConway‐Maxwell Poisson regression model with multicollinearity: Methods and applications. *Concurrency and Computation: Practice and Experience* .2021; 10.1002/cpe.6477 10.1002/cpe.6477

[4] : A new adjusted Liu estimator for the Poisson regression model. *Concurrency and Computation: Practice and Experience* .2021; 10.1002/cpe.6340 10.1002/cpe.6340

[5] : On the Liu estimation of Bell regression model in the presence of multicollinearity. *Journal of Statistical Computation and Simulation* .2021; 10.1080/00949655.2021.1955886 1-21 10.1080/00949655.2021.1955886

[6] : A New Ridge Estimator for the Poisson Regression Model. *Iranian Journal of Science and Technology, Transactions A: Science* .2019;43(6) : 10.1007/s40995-019-00769-3 2921-2928 10.1007/s40995-019-00769-3

[7] : Adjusted Adaptive LASSO in High-dimensional Poisson Regression Model. *Modern Applied Science* .2015;9(4) : 10.5539/mas.v9n4p170 10.5539/mas.v9n4p170

[8] : DIAGNOSTIC IN POISSON REGRESSION MODELS. *Electronic Journal of Applied Statistical Analysis* .2012;5(2) :178-186

[9] : Proposed methods in estimating the ridge regression parameter in Poisson regression model. *Electronic Journal of Applied Statistical Analysis* .2018;11(2) : 10.1285/i20705948v11n2p506 505-515 10.1285/i20705948v11n2p506

# Peer review: Reviewer response for version 1

**DOI:** 10.5256/f1000research.57427.r89260

**Published:** 2021-07-21

**Authors:** Mohammad Arashi

**Affiliations:** 1 Department of Statistics, Faculty of Mathematical Sciences, Ferdowsi University of Mashhad, Mashhad, Iran

The paper extends the Liu estimator in generalized linear modeling. Specifically, the authors propose a new biased estimator for the estimation of regression coefficients in the discrete Poisson regression.

 The results are interesting and the topic is eye-catching. The theoretical results are well supported by extensive numerical analysis.

 I suggest the authors make minor revisions to improve the presentation before indexing.

1. Check the notation entirely to be consistent. For instance, in equation (2.10), "hat" must be added for the estimator. It happens also for (2.12).
2. Use another notation for diagonal matrices in equations. For example, you may use "L" and then define the elements. 
3. Explain equation (2.21) more. Is the minimization over i?
4. In the simulation study, for the design generation, I suggest using another notation for "z _ip_" since it is not the last element of the series of generated independent normals.
5. Provide a reference for the accessibility of the real data use in the real-life application.

Is the work clearly and accurately presented and does it cite the current literature?

Yes

If applicable, is the statistical analysis and its interpretation appropriate?

Yes

Are all the source data underlying the results available to ensure full reproducibility?

Partly

Is the study design appropriate and is the work technically sound?

Yes

Are the conclusions drawn adequately supported by the results?

Yes

Are sufficient details of methods and analysis provided to allow replication by others?

Yes

Reviewer Expertise:

High-dimensional modeling; shrinkage estimation

I confirm that I have read this submission and believe that I have an appropriate level of expertise to confirm that it is of an acceptable scientific standard, however I have significant reservations, as outlined above.

# Response

**Published:** 2021-07-27

**Authors:** BENEDICTA Aladeitan

**Affiliations:** Landmark University , Nigeria

Thanks for your observations and corrections. All will be duely implemented.
